# Vestibular evoked myogenic potentials in affected and asymptomatic ears in unilateral Ménière's Disease

**DOI:** 10.1016/S1808-8694(15)31286-6

**Published:** 2015-10-20

**Authors:** Súnia Ribeiro, Roberta R. de Almeida, Heloisa H. Caovilla, Maurício M. Ganança

**Affiliations:** 1 Physician, Master studies under course, Discipline of Otoneurology, Federal University of Sao Paulo – Escola Paulista de Medicina; 2 Ph.D. in Medicine (Major in Otorhinolaryngology), Medical School, University of Sao Paulo. Assistant Physician, Discipline of Otoneurology, Federal University of Sao Paulo – Escola Paulista de Medicina; 3 Associate Professor, Full Professor, Discipline of Otoneurology, Federal University of Sao Paulo – Escola Paulista de Medicina; 4 Faculty Professor of Otorhinolaryngology, Federal University of Sao Paulo – Escola Paulista de Medicina

**Keywords:** evoked potentials, vestibular function test, Ménière's disease/diagnosis

## Abstract

**Aim:**

To verify whether vestibular evoked myogenic potentials can present abnormalities in the affected ear and in the asymptomatic ear in patients with diagnosis of unilateral Ménière's disease.

**Study design:**

Transversal cohort.

**Material and Method:**

The vestibular evoked myogenic potentials of 20 patients with unilateral Ménière's disease were analyzed. The selection of individuals was based on the history and in clinical evaluation suggestive of unilaterally defined Ménière's disease, and with electrocochleography abnormalities in the affected ear. Vestibular evoked myogenic potentials were evaluated in both ears of each patient through absolute latencies of p13 and n23, interaural difference of latency of peaks p13 and n23 and amplitude p13-n23 asymmetry rate.

**Results:**

Vestibular evoked myogenic potentials were altered in 35.0% of the affected ears and in 25.0% of the asymptomatic ears. The alterations were: absence of responses in seven cases, prolongation of p13 latency in three cases, and increase in interaural amplitude difference ratio in one case.

**Conclusion:**

The vestibular evoked myogenic potentials can present abnormalities in the affected and asymptomatic ears in patients with diagnosis of unilaterally defined Ménière's disease.

## INTRODUCTION

Ménière's disease was described by Prosper Ménière in 1861 and it was defined as a membranous labyrinth disease, characterized by recurrent spontaneous episodes of vertigo, hearing loss, ear fullness and tinnitus on the affected side owing to endolymphatic hydrops (American Academy of Otolaryngology – Head and Neck Surgery, 1995)^1^.

Endolymphatic hydrops is most frequently found in the cochlea and sacculus is the second most prevalent site of affection. Severe hydrops is more common in the sacculus^2^. Saccular hydrops may occur in 50% of the cases of Ménière's syndrome^3^. The definition of saccular hydrops may represent an important breakthrough in the diagnosis of Ménière's disease^4^, ^5^, ^6^, ^7^, ^8^.

One hundred and fifty years later, many aspects concerning etiopathogenesis and therapy of Ménière's disease are still controversial. From a diagnostic perspective, many tests have been exhaustively studied, but the questions still persist.

The presence of in vivo hydrops has never been confirmed, but it has been suggested by the results of glycerol or furosemide tests and by electrocochleography. However, these tests are not appropriate to assess otolithic organs or descending neural pathways (lateral vestibular-spinal tract). New clinical instruments are necessary to identify saccular hydrops.

VEMP – Vestibular Evoked Myogenic Potentials have been studied since the 60's, but many different centers started to use it to assess the sacculocollic reflex after 1992. They are middle-latency evoked potentials generated by vestibular-spinal muscle reflex that depend on functional integrity of sacculus macula, inferior vestibular nerve, vestibular nuclei, vestibular-spinal pathways and neuromuscular plates^9^. Damage to any of these structures results in affection of potentials. Prolongation of latency suggests retrolabyrinthic damage, especially of the vestibular-spinal tract. In dysfunctions of saccular macula or inferior vestibular nerve, the affection described is asymmetry of amplitude or absence of response in the affected side, showing reflex blockage^10^.

The recording of the first complex of biphasic wave p13-n23 presents positive peak (p) with middle latency of 13ms, followed by negative peak (n) with middle latency of 23ms. Amplitude of p13-n23 expresses the magnitude of muscle reflex generated by sound stimulation of sacculus macula^11^.

In unilateral Ménière's disease, absence of vestibular myogenic potentials was observed in 54.2% of the cases^12^; reduced absence or amplitude of potentials was identified in 58.8% of the patients^5^.

Abnormal responses were detected in 53.3% of affected ears and in 6.6% of asymptomatic ears of patients with unilateral Ménière's disease^6^.

The increase in interaural difference of amplitude of potentials suggests sacculus hydrops in unilateral Ménière's disease^7^.

Many studies have shown the value of vestibular evoked myogenic potentials in the assessment of saccular functions^4^, ^7^, ^8^, ^10^, ^13^. The investigation of these potentials is a non-invasive objective procedure easy to perform and interpret that does not result in patients' discomfort.

Asymptomatic ears in patients with unilateral Ménière's disease may evidence similar abnormalities to the affected ears, but at lower levels of intensity. This finding may be resultant from occult sacculus hydrops in the asymptomatic ear or from binaural interactions in otolithic-cervical reflex arch of vestibular evoked myogenic potentials^14^.

The purpose of the present study was to check whether vestibular evoked myogenic potentials could present abnormalities in the affected ear and in the asymptomatic ear in patients with diagnostic hypothesis of unilateral Ménière's disease.

## MATERIAL AND METHOD

The research project was assessed and approved by the Research Ethics Committee, Hospital Sao Paulo – Escola Paulista de Medicina (UNIFESP).

We assessed vestibular evoked myogenic potentials in 20 patients with unilateral Ménière's disease, according to clinical criteria set forth by the American Academy of Otolaryngology – Head and Neck Surgery (1995); there were 16 female and four male subjects, ages ranging from 30 to 82 years, mean age of 49.5 years.

The selection of subjects was based on clinical history and assessment suggestive of unilaterally defined Ménière's disease and electrocochleography with abnormalities in the affected ear.

The studied subjects were submitted to ENT and otoneurological exams, including pure tone and vocal audiometry, immittanciometry, transtympanic electrocochleography, brainstem audiometry, vectroelectronystagmography, and vestibular evoked myogenic potentials.

Transtympanic electrocochleography, conducted based on Munhoz (2001)^15^ criteria, suggested cochlear endolymphatic hydrops when the ratio between summation potential and action potential (PS/PA) was greater or equal to 35.0%.

Exclusion criteria were: 1) deviation from normal range hearing threshold, vocal discrimination, immittanciometry and transtympanic electrocochleography of asymptomatic ear; 2) inability to turn the head; and 3) deformity of ear pinna, external ear canal our tympanic membrane.

The equipment used was four-channel Amplaid MK12^R^. Sound stimuli were presented by headsets Amplaid MX-41/AR^R^.

The recordings were made by surface electrodes placed over the skin and fixed with adhesive tape, after previous use of electrolytic paste. The active electrode was placed on the upper half of sternocleidomastoid muscle ipsilateral to stimulation; reference electrode, over the anterior border of ipsilateral clavicle, and ground electrode, on the frontal midline.

**Figure 1:**
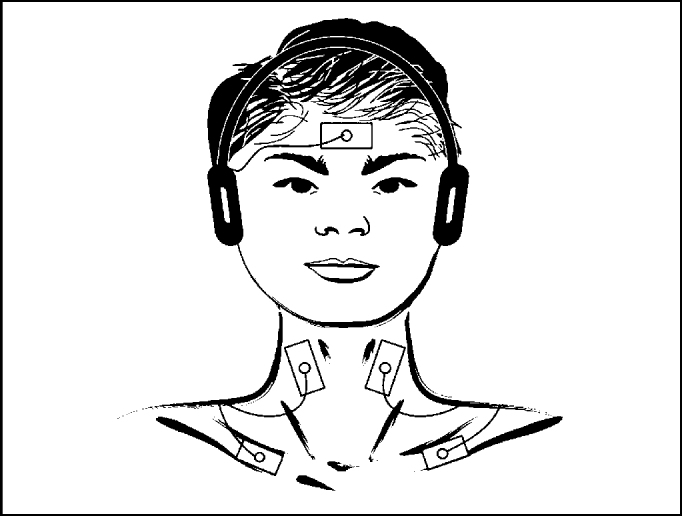
Sites of electrode placement to record responses from sternocleidomastoid muscle.

**Figure 2:**
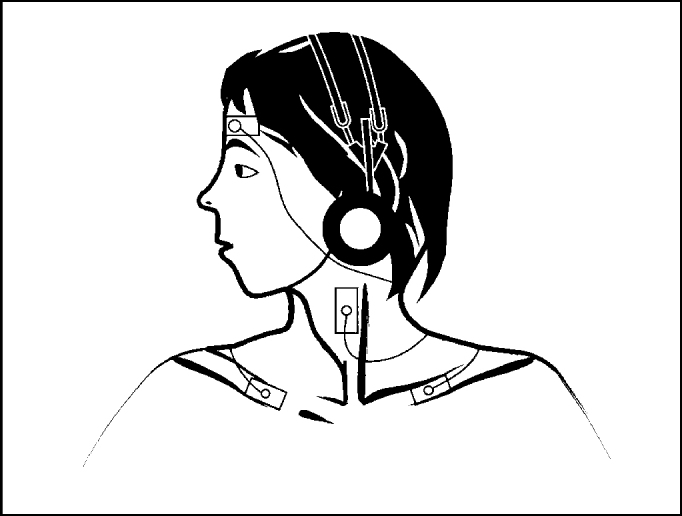
Position of subject during recording of responses from sternocleidomastoid muscle.

To obtain vestibular evoked myogenic potentials from sternocleidomastoid muscle, the patient remained seated, with maximum head lateral rotation to the side contralateral to the stimulus. The stimulus was started by right side afferent input and then repeated on the left. The responses were replicated, that is, recorded twice on the right side and twice on the left side (Figures 1 and 2).

The tests were conducted in silent but not acoustically treated environment.

We conducted promeasurement of 200 clicks alternated with 0.1ms duration, frequency of presentation of 3Hz, intensity of 90dB HL (120 dB SPL), using pass band filter of 10 to 2500 Hz, with amplification of 10 to 25 μV by division. The recordings were conducted within a window of 60ms.

**Figure 3:**
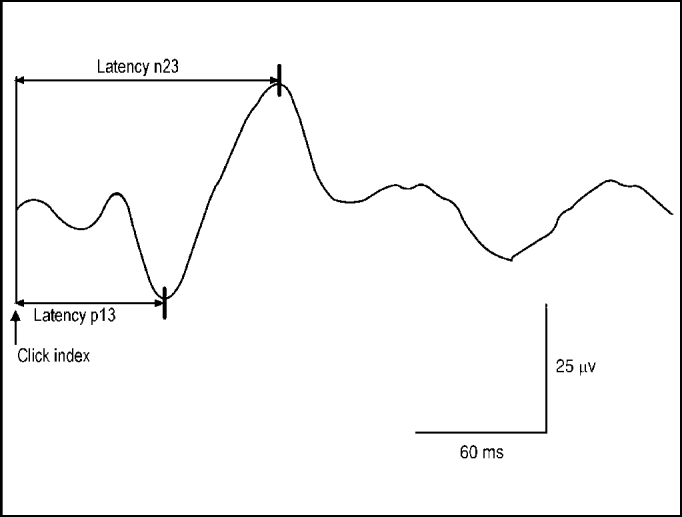
Complex of biphasic wave p13-n23.

Figure 3 shows the recording of the first complex of biphasic waves p13-n23, with positive peak followed by negative peak.

Vestibular evoked myogenic potentials were assessed by absolute latencies p13 and n23, interaural difference of latency in peaks p13 and n23, and amplitude p13-n23 asymmetry rate.

The interaural difference of latency in peaks p13 and n23 was calculated by a formula in which the value of the highest latency side was subtracted from the value of the lowest latency side. Based on data from normal patients seen by Almeida (1999)^16^, which employed the same equipment and method, absolute latency of peak p13 was considered abnormal when it exceeded 15.82 ms.

We used rate of interaural amplitude asymmetry of vestibule-spinal muscle reflex, expressed in percentage, calculated by the formula: 100 (side of highest amplitude – side of lowest amplitude)/(side of highest amplitude + side of lowest amplitude). Based on values of normal subjects by Almeida (1999)^16^, level of amplitude asymmetry was considered abnormal when it exceeded 44.4%.

To assess vestibular evoked myogenic potentials we compared the analysis of affected ear and asymptomatic ear.

T-paired test was applied to check whether there was difference between the means of values of two paired groups with numeric measurement. The level of significance was 5%.

## RESULTS

Ten patients with unilateral Ménière's disease (50.0%) did not show any abnormality in vestibular evoked myogenic potentials.

Ten cases (50.0%) presented abnormalities. Potentials were absent in seven cases (35%); two cases presented absence of bilateral responses, and five cases had unilateral absence, with three cases of affected side and two cases of asymptomatic side. Increase in absolute latency of p13 was identified in two cases (10%), and association of increased absolute latency of p13 and amplitude asymmetry rate in one case (5%) (Table 1).

**Table 1:** Descriptive analysis of abnormal results in Vestibular evoked myogenic potentials in patients with unilateral Ménière's disease.

ABNORMAL RESPONSES	N	%
Absence of bilateral response	2	10
Absence of response on the symptomatic side	3	15
Absence of response on the asymptomatic side	2	10
Increase in absolute latency of p13 on the symptomatic side	1	5
Increase in absolute latency of p13 on the asymptomatic side	1	5
Increase in absolute latency of p13 and increase in asymmetry rate (> ipsilateral response)	1	5
TOTAL	10	50

Vestibular Evoked Myogenic Potentials on the asymptomatic ear side did not present abnormalities in 15 cases (75%), it was absent in four cases (20%), and evidenced increase in absolute latency of p13 in one case (5%).

In the 13 cases (65%) with presence of Vestibular Evoked Myogenic Potentials, the mean of p13 potential latency to stimulation ipsilateral to sternocleidomastoid muscle of the affected ear was 12.26 ± 1.94ms, and the mean of n23 potential latency was 20.17 ± 1.53ms. Latencies p13 and n23 of asymptomatic ears were 11.98 ± 1.69ms and 20.01 ± 1.99ms, respectively. There were no statistically significant differences between means of affected ear and means of asymptomatic ear (Table 2).

**Table 2:** Results of the comparison of latency values of Vestibular evoked myogenic potentials between asymptomatic and affected ears.

		Asymptomatic ear	Affected ear	T-paired test (p)
	Mean	11.98	12.26	
L p13 (ms)	Standard deviation	1.69	1.94	0.684
	N	13	13	
	Mean	20.01	20.17	
L n23 (ms)	Standard deviation	1.99	1.53	0.818
	N	13	13	

Key: L = latency

p13 = positive peak with 13ms latency

n23 = negative peak with 23ms latency

Values of absolute latency p13 above 15.82ms were found in three patients (15%), two on the affected side and one on the asymptomatic side.

There was no statistically significant difference between mean of absolute values of amplitude p13-n23 in 13 affected ears, equal to 29.69 ± 26.35μv, and asymptomatic ears, equal to 30.98 ± 24.75μv (Table 3).

**Table 3:** Results of the comparison of values of amplitude of Vestibular evoked myogenic potentials between the asymptomatic and affected ear.

	Asymptomatic ear	Affected ear	T-paired test (p)
Mean	30.98	29.69	
A p13-n23 (uV) Standard deviation	24.75	26.35	0.786
N	13	13	

Key: A = amplitude

p13 = positive peak with 13ms latency

n23 = negative peak with 23ms latency

To conduct statistical analysis of absolute latencies and amplitudes, we excluded absence of response (7 cases), so the sample comprised 13 ears.

The rate of amplitude asymmetry was 26.77 ± 12.65% (Table 4).

**Table 4:** Values of interaural differences of p13 and n23 and asymmetry rate.

	Interaural Difference p13	Interaural Difference n23	Asymmetry Rate (%)
Mean	1.72	1.75	26.77
Standard deviation	1.62	1.68	12.65
Median	1.44	0.96	27.07
Minimum	0.24	0.18	0.68
Maximum	5.76	4.80	48.20
N	13	13	13

Key:

p13 = positive peak with 13ms latency

n23 = negative peak with 23ms latency

One of the patients with increase in rate of amplitude asymmetry presented increase in absolute latency of p13 on the affected ear.

## DISCUSSION

The diagnostic difficulties of defining Ménière's disease, which can only be made through histological analysis after death, have led to the development of clinical criteria to understand the diagnostic hypothesis of this labyrinthic affection. These criteria are based on the association of clinical signs and symptoms with otoneurological findings^17^.

Electrocochleography associated or not with glycerol or furosemide tests is one of the most employed exams for the diagnosis of endolymphatic hydrops. The literature on the topic addresses ideal parameters, the importance of identifying endolymphatic hydrops and specificity or sensitivity in diagnosing Ménière's disease^17^, ^18^, ^19^. Sacculus is the second most prevalent area of hydrops^2^. Electrocochleography is not appropriate for the assessment of otolithic organs (sacculus, utriculus and semicircular canals) or descending neural pathways. The determination of saccular hydrops may be useful in the diagnosis of Ménière's disease^4^, ^5^, ^6^, ^7^, ^8^.

Vestibular evoked myogenic potentials is a test used to assess sacculocollic reflex in humans, triggered by intensive sound stimulation of saccular macula, which is sensitive to sound even with total destruction of the cochlea^20^. These potentials may be obtained with the same stimulus as in subjects with poor or good auditory acuity, given that sacculus macula is intact.

In electrocochleography, auditory thresholds are the limiting factor of the exam. Munhoz (2001)^15^ noticed that the value of 35% for PS/PA ratio in patients with hearing thresholds d•50dB corresponded to a rate of 100% of sensitivity and specificity to characterize endolymphatic hydrops. Soares et al. (2003)^21^, however, upon studying patients with auditory thresholds e•50dB, observed that electrocochleography has less sensitivity and specificity.

Integrity of sacculocollic reflex in patients with Ménière's disease is confirmed by the presence of biphasic wave p13-n23 in Vestibular evoked myogenic potentials. Biphasic wave p13-n23 occurred on the affected ear in 15 (75%) of the patients with unilateral Ménière's disease. This result was similar to that reported by Seo et al. (2003)^8^ who found biphasic wave in 18 cases (72%), but it is in disagreement with Waele et al. (1999)^12^, who observed wave p13-n23 in only 23 (45.7%) cases.

Waele et al. (1999)^12^ attributed the absence of response to Vestibular evoked myogenic potentials in Ménière's disease as resulting from insufficient muscle contraction during the test, occult peripheral affection or sacculus hyposensitivity, owing to aging of saccular macula in the elderly. In our study, myogenic potentials were absent in seven cases (35%). Absence of response to Vestibular evoked myogenic potentials in cases of Ménière's disease suggests saccular hydrops^8^, ^10^, ^12^. Depending on the severity of hydrops, some patients may present irreversible degeneration of sensorial epithelium of saccular macula^5^, ^6^, ^8^, ^13^. Absence of response on the non-affected ear was found in 20% of our cases. Similar findings were detected in the study by Seo et al. (2003)^8^ on six ears (24.0%) of 25 patients, showing the value of these potentials in the diagnosis of occult saccular hydrops, without clinical manifestations^14^.

Some authors have reported abnormal findings in clinical exams of the asymptomatic ear in Ménière's disease. Endolymphatic hydrops may be evidenced in electrocochleography of asymptomatic ears in 15.0 to 35.0% of patients with unilateral Ménière's disease^22^, ^23^. Histopathological confirmation of endolymphatic hydrops of the asymptomatic ear was found in 11.1% of the cases by Fraysse et al. (1989)^24^. These findings indicated that electrophysiological exams could identify occult endolymphatic hydrops on ears that were apparently asymptomatic in patients with Ménière's disease. In our study, we noticed absence of absolute latency in p13 in 3 cases (15.0%). Similar findings were detected in the study by Young et al. (2002b)^25^, reporting 10% of cases of late endolymphatic hydrops with prolongation of absolute latency of p13, justifying these findings owing to high endolymphatic pressure that would affect the transmission of sound, provided that hearing was also affected. To Murofushi et al. (2001a)^10^, prolongation of latency of p13 suggested retrolabyrinthic damage.

We did not find statistically significant difference between absolute latencies of waves p13 and n23 on affected and asymptomatic ears. The same was found by Young et al. (2003)^26^. Waele et al. (1999)^12^ observed latency of response in subjects with Ménière's disease similar to that of normal subjects, detecting differences only in amplitude of response.

Amplitude of the first biphasic potential was considered as a quantitative measurement of vestibular-spinal reflex, expressing electrical activity of effector muscles^12^, ^27^, ^28^, ^29^, ^30^. The variability of responses is due to individual differences in level of contraction, tone and mass of the studied muscles, despite standardization of posture of the patient during the conduction of the exam. Thus, absolute amplitude should not be used for interpersonal comparison of vestibular-spinal muscle reflex but rather as amplitude asymmetry rate. The increase in rate of asymmetry amplitude of potentials is suggestive of saccular hydrops in unilateral Ménière's disease^7^.

As to responses to sternocleidomastoid muscle in ipsilateral monoaural stimulation, we confirmed that the mean of asymmetry rate of patients that presented bilateral responses was 26.77% with standard deviation of 12.65%. The literature describes normal responses with asymmetry rate of up to 34% (Murofushi et al., 1998)^29^, 36.0% (Young et al., 2002a)^7^, 44.4% (Almeida, 1999)^16^ and 47.4% (Seo et al., 2003)^8^. In our study, we found one patient (5%) with increase in Vestibular evoked myogenic potentials that presented rate of amplitude asymmetry of 48.2%, suggesting hypersensitivity of saccular macula caused by hydrops of the organ. Similar result was found in the literature in 5 to 7.5% of the cases^7^, ^26^. Conversely, in seven patients we observed absence of ipsi or contralateral responses on the affected side, suggesting arreflexia of saccular macula and, therefore, a more advanced stage of the disease in the organ.

In our study, Vestibular evoked myogenic potentials were affected in 50% of the patients with unilateral Ménière's disease, which is close to the results presented by Waele et al. (1999)^12^, Murofushi et al. (2001b)^5^, Shojaku et al. (2001)^6^, Seo et al. (2003)^8^ and Young et al. (2003)^26^, between 40.0 and 54.0%.

Based on what we could observe, Vestibular evoked myogenic potentials provide information that may be useful in the assessment of involvement of sacculus in endolymphatic hydrops of Ménière's disease, both on the affected and on the asymptomatic ear. Despite the fact that the assessment takes into account the diagnosis of Ménière's disease as unilateral through cochlear criteria (clinical and electrocochleography), what we could observe is that the disease impairs differently the many labyrinthic segments, reason why there is so much heterogeneity of response in the so-called unilateral disease.

Easiness to conduct and interpret, the fact it is not invasive and does not cause discomfort in patients are advantages of this method to be considered.

Studies that appreciate the implications of Vestibular evoked myogenic potentials in different stages of Ménière's disease and in other forms of endolymphatic hydrops are necessary. We believe that the method deserves to be included in the clinical routine of otoneurological assessment.

## CONCLUSION

Vestibular evoked myogenic potentials may present abnormalities on the affected and asymptomatic ears in patients with diagnostic hypothesis of unilaterally defined Ménière's disease.
